# Association between serum phosphate levels and outcome among critically ill newborns: a retrospective cohort study

**DOI:** 10.3389/fped.2026.1909660

**Published:** 2026-08-27

**Authors:** Lisha Bao, Xiaohui Liu, Caixia Zhao, Jing Li, Dan Li, Zhifang Du

**Affiliations:** 1 Neonatal Department, Bethune International Peace Hospital, Shijiazhuang, China; 2 Neonatal Department, Shijiazhuang Maternal and Child Health Hospital, Shijiazhuang, China; 3 Pharmacy Department, Bethune International Peace Hospital, Shijiazhuang, China

**Keywords:** 28-day mortality, hyperphosphatemia, hypophosphatemia, in-hospital death, length of hospital stay (hospital LOS), newborn, outcome, phosphate

## Abstract

**Background:**

This study aimed to explore the relationship between serum phosphate levels and clinical outcomes in critically ill neonates.

**Methods:**

A retrospective cohort study was conducted using the Pediatric Intensive Care (PIC) database from (2010- 2018.) Multivariable regression models, generalized additive models, and threshold effect analysis were adopted to evaluate the relationship between serum phosphate levels within 24 h of ICU admission (Pi24) and neonatal outcomes.

**Results:**

A total of 1,902 critically ill neonates were enrolled. Pi24 was stratified into three tertile groups (Q1–Q3). Baseline characteristics and Kaplan–Meier curves showed that higher Pi24 correlated with higher 28-day mortality, peaking at 11% in the Q3 group. Multivariate Cox proportional hazards regression analysis revealed that, Q3 neonates had a 76% increased risk of 28-day mortality compared with Q1(HR = 1.76, 95%CI:1.08∼2.88, *P* = 0.025), Multivariate logistic regression indicated an 80% higher odds of in-hospital death in Q3 vs. Q1(OR = 1.80, 95%CI: 1.1–2.95, *P* = 0.019). Within Pi24 ranged from 1.76 to 4.37 mmol/L, each 1.0 mmol/L elevation in serum phosphate level increased in-hospital death risk by 45.6% (OR = 1.456, 95% CI: 1.039–2.039, *P* = 0.029). Multivariate linear regression analysis revealed that Q1 neonates had significantly longer hospital LOS than Q3 (*β* = −4.68, 95% CI: −7.84∼−1.53, *P* = 0.004). Threshold effect analysis further demonstrated that when serum phosphate was < 2.5 mmol/L, each 1.0 mmol/L reduction prolonged hospital LOS by 6.89 days (*β* = −6.892, 95% CI: −10.801∼−2.983, *P* = 0.001).

**Conclusion:**

Elevated Pi24 levels were associated with increased risks of 28-day mortality and in-hospital death in critically ill neonates, while lower Pi24 levels independently predict prolonged hospital LOS Pi24 may represent a valuable prognostic biomarker for this population.

## 1 Introduction

Critically ill patients frequently experience disturbances in the internal environment accompanied by metabolic abnormalities involving electrolytes and minerals, including sodium, potassium, chloride, calcium, and phosphorus. Among these abnormalities, disorders of serum phosphate metabolism are often overlooked. Intracellular phosphate plays an essential role in numerous cellular processes, including signal transduction, nucleic acid synthesis, membrane integrity, and energy metabolism. In addition, phosphorus is a crucial component of 2,3-diphosphoglycerate (2,3-DPG) in erythrocytes and contributes to oxygen transport. Phosphate also plays a vital role in maintaining acid-base homeostasis in plasma and urine (1). Therefore, phosphorus is indispensable for normal physiological functions, and phosphate metabolic disorders may adversely affect the prognosis of critically ill newborns. In recent years, numerous studies have explored the relationship between serum phosphate levels and clinical outcomes in critically ill adult and pediatric patients, yet the conclusions remain inconsistent (2, 3).

Neonates represent a unique population characterized by immature organ function and distinct physiological characteristics compared with older children and adults. Serum phosphate concentrations are physiologically highest in the neonatal period and gradually decline to the typical adult level with age (4). Given the specificity of phosphate metabolism in neonates, the impact of phosphate metabolic disturbances on clinical outcomes in critically ill neonates may differ from that observed in adults and older children. However, evidence regarding the association between serum phosphate levels, disease severity and clinical prognosis in critically ill neonates remains limited.

To further investigate the relationship between phosphate metabolism and prognosis in critically ill neonates, the present study aimed to evaluate the associations between serum phosphate levels within 24 h of admission (Pi24) and clinical outcomes, including 28-day mortality, in-hospital death, and hospital length of stay (LOS), in this population.

## 2 Materials and methods

### 2.1 Database

This was a retrospective observational cohort study based on data from the Pediatric Intensive Care (PIC) database. The PIC database is a large, publicly accessible, specialized single-center pediatric database (5). It contains data from 12,881 pediatric patients with 13,941 hospitalization records admitted to the intensive care units of the Children's Hospital, Zhejiang University School of Medicine, between 01/01/2010 and 31/12/2018.

This study was approved by the Institutional Review Board of the Children's Hospital, Zhejiang University School of Medicine, Hangzhou, China, and the Medical Ethics Committee of Bethune International Peace Hospital (Approval No. 2026-KY-65). Since this retrospective study did not involve any clinical intervention, the requirement for informed consent was waived.

### 2.2 Study population

This study included critically ill neonates with a postnatal age ≤ 28 days who were admitted to the intensive care units of the Children's Hospital of Zhejiang University School of Medicine between 2010 and 2018. Preterm birth was identified according to the International Classification of Diseases, 10th Revision (ICD-10) codes.

The inclusion criteria were as follows: (1) Postnatal age ≤ 28 days; (2) Available serum phosphate measurement within 24 h of admission (Pi24); (3) First admission to the ICU.

No restriction was applied regarding the timing of in-hospital death.

### 2.3 Exposure variable

The exposure variable was serum phosphate concentration within 24 h of ICU admission (Pi24). When multiple serum phosphate measurements were available within this time window, the mean value was used for analysis.

### 2.4 Data extraction and covariates

Based on previous literature, the following covariates were collected: postnatal age, gender, preterm/term delivery, 28-day mortality, in-hospital death, hospital LOS, comorbid sepsis, gastrointestinal malformations, congenital heart disease (CHD), intracranial hemorrhage (ICH), neonatal asphyxia, acute kidney injury (AKI), and acute liver injury (ALI).

Laboratory parameters measured within 24 h of admission included white blood cell count (WBC), platelet count, hemoglobin, serum total calcium (Ca), prealbumin, alanine transaminase (ALT), creatine kinase isoenzyme MB (CK-MB), blood urea nitrogen (BUN), creatinine (Cr), and lactate (LAC)

Comorbidities were identified according to ICD-10 codes. AKI was defined as an increase in serum creatinine by ≥ 26.5 *μ*mol/L within 48 h or an increase of 150%–199% from the baseline value (6). ALI was defined as an ALT level exceeding twice the age-specific upper limit of normal (7). Covariates with a missing rate over 40% were excluded. No missing data were observed among the covariates included in the present study.

### 2.5 Outcome measures

The primary outcomes included 28-day mortality, in-hospital death, and hospital LOS.

### 2.6 Statistical analysis

Baseline characteristics were compared among groups stratified by two grouping methods: Pi24 tertiles and cut-off values of 1.6 mmol/L and 2.6 mmol/L (8, 9). Continuous variables were presented as mean ± standard deviation or median with interquartile range, while categorical variables were presented as frequencies and percentages. Differences among groups were assessed using the chi-square test or Fisher's exact test for categorical variables, one-way analysis of variance (ANOVA) for normally distributed continuous variables, and the Kruskal–Wallis H test for skewed-distributed variables.

Univariate regression analyses were performed to evaluate the associations between covariates and outcomes in critically ill neonates. Multivariate Cox proportional hazards, logistic, and linear regression models were subsequently applied to investigate the associations between Pi24 and 28-day mortality, in-hospital death, and hospital LOS, respectively. Covariates with statistical significance in univariate analysis (*P* < 0.05) and those with clinical relevance were included in the multivariate models. All analyses were conducted in accordance with the Strengthening the Reporting of Observational Studies in Epidemiology (STROBE) statement (10). The initial analyses were performed using unadjusted models.

The multivariate Cox proportional hazards regression model for 28-day mortality and the logistic regression model for in-hospital death were adjusted for postnatal age, sex, preterm birth, neonatal asphyxia, ALI, AKI, prealbumin, WBC, platelet count, CK-MB, and LAC. Interaction and subgroup analyses were further performed according to sex, preterm birth status, congenital heart disease, gastrointestinal malformations, and ALI.

The multivariate linear regression model for hospital LOS was adjusted for postnatal age, gender, preterm birth, neonatal asphyxia, sepsis, gastrointestinal malformations, hemoglobin, WBC, platelet count, CK-MB, BUN, prealbumin, and LAC.

Smooth curve fitting was performed to explore potential nonlinear associations between Pi24 and clinical outcomes, including 28-day mortality, in-hospital death, and hospital LOS. When a significant nonlinear association was identified, piecewise linear regression analysis was conducted to further quantify the threshold effect of serum phosphate levels on these outcomes based on the fitted smooth curves (11).

Subgroup analysis and sensitivity analyses were conducted to assess the robustness of the findings. Subgroup analyses were performed according to premature, CHD, digestive tract malformation, AKI, and ALI. However, subgroup analyses evaluating the associations of Pi24 with 28-day mortality and in-hospital death were not stratified by AKI, because the limited sample size within each subgroup could compromise the reliability of statistical outcomes. Sensitivity analyses were conducted after excluding infants who died within 24 h of admission, those with congenital hereditary diseases or tumors, and those with AKI.

All statistical analyses were performed using R software version 3.3.2 (http://www.R-project.org, R Foundation) and Free Statistics software version 2.2. A two-tailed *P*-value < 0.05 was considered statistically significant.

## 3 Results

### 3.1 Participants selection

**Figure 1:**
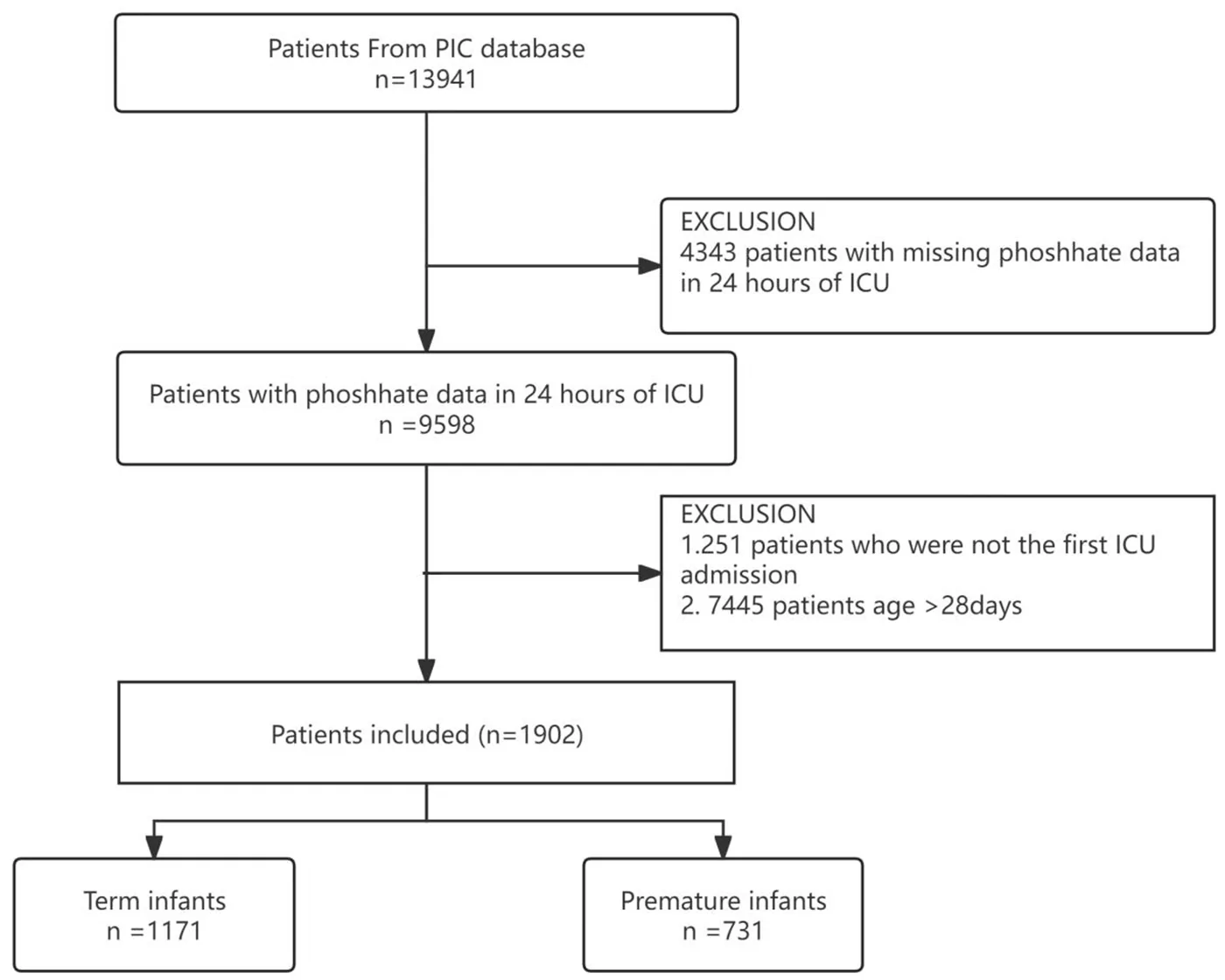
Flowchart of patient selection.

Among the total 13,941 hospitalization records extracted from the PIC database, 4,343 cases without serum phosphate detection data within 24 h after admission, 251 cases with non-first ICU admission, and 7,445 cases with postnatal age over 28 days were excluded. Finally, a total of 1,902 critically ill neonates were included in the present study, comprising 1,171 full-term neonates and 731 preterm neonates (Figure 1).

### 3.2 Baseline characteristics

Baseline characteristics of all enrolled participants are presented in Table 1. Participants were categorized into three groups according to Pi24 tertiles: the low Pi24 group (Q1, 0.34–1.90 mmol/L), intermediate Pi24 group (Q2, 1.91–2.48 mmol/L), and high Pi24 group (Q3, 2.49–6.68 mmol/L). Baseline characteristics stratified by previously reported Pi24 cut-off values of 1.6 and 2.6 mmol/L are presented in Supplementary Table S1, including the hypophosphatemia group (<1.6 mmol/L), normal phosphate group (1.6–2.6 mmol/L), and hyperphosphatemia group (>2.6 mmol/L). The prevalence of hypophosphatemia and hyperphosphatemia was 15.7% (299/1,902) and 28.5% (543/1,902), respectively.

**Table 1:** Baseline characteristics of participants.

Co variate	Total	Phosphate level(mmol/L)			*P*-value
		Q1Low (0.34-1.9)	Q2Mild (1.91-2.48)	Q3High (2.49–6.68)	
	(*n* = 1,902)	(*n* = 634)	(*n* = 633)	(*n* = 635)	
Baseline characteristics					
Gender,males *n* (%)	1,164 (61.2)	380 (59.9)	397 (62.7)	387 (60.9)	0.589
Age,days	1.0 (0.0, 7.0)	4.0 (1.0, 14.0)	1.0 (0.0, 6.0)	1.0 (0.0, 2.0)	<0.001
Premature, *n* (%)	731 (38.5)	233 (36.8)	248 (39.3)	250 (39.4)	0.551
Outcome					
28-day Mortality,n(%)	148 (7.8)	34 (5.4)	44 (7)	70 (11)	<0.001
In-hospital death,n (%)	172 (9.0)	41 (6.5)	53 (8.4)	78 (12.3)	0.001
Hospital.LOS, days	20.0 (10.0, 41.0)	23.0 (12.0, 44.0)	20.0 (10.0, 39.0)	16.0 (8.0, 38.5)	<0.001
Comorbidities					
CHD, *n* (%)	231 (12.1)	67 (10.6)	95 (15)	69 (10.9)	0.026
Neonatal asphyxia, *n* (%)	201 (10.6)	59 (9.3)	53 (8.4)	89 (14)	0.002
Sepsis, *n* (%)	82 (4.3)	38 (6)	23 (3.6)	21 (3.3)	0.036
Digestive tract anomaly, *n* (%)	217 (11.4)	116 (18.3)	49 (7.8)	52 (8.2)	<0.001
ICH, *n* (%)	51 (2.7)	15 (2.4)	20 (3.2)	16 (2.5)	0.651
ALI, *n* (%)	72 (3.8)	25 (3.9)	20 (3.2)	27 (4.3)	0.576
AKI, *n* (%)	59 (3.1)	11 (1.7)	17 (2.7)	31 (4.9)	0.004
Laboratory examination					
Phosphate, mmol/L	2.4 ± 0.9	1.6 ± 0.3	2.2 ± 0.2	3.4 ± 0.8	<0.001
TotalCa,mmol/L	2.1 ± 0.3	2.1 ± 0.3	2.1 ± 0.3	2.1 ± 0.4	0.01
Prealbumin, g/L	0.1 ± 0.0	0.1 ± 0.0	0.1 ± 0.0	0.1 ± 0.0	0.254
ALT,U/L	11.0 (7.0, 18.0)	11.0 (6.0, 21.0)	10.0 (6.0, 15.8)	11.0 (7.0, 17.0)	0.009
BUN, mmol/L	5.1 ± 3.2	4.7 ± 3.0	4.9 ± 3.4	5.8 ± 3.2	<0.001
Cr, µmol/L	80.4 ± 34.5	68.1 ± 30.7	79.0 ± 31.2	94.3 ± 36.3	<0.001
LAC,mmol/L	2.6 (1.7, 4.1)	2.0 (1.4, 3.4)	2.7 (1.8, 4.1)	3.0 (2.0, 4.5)	<0.001
Hemoglobin,g/L	146.6 ± 37.7	132.7 ± 35.5	149.0 ± 35.5	158.2 ± 37.6	<0.001
WBC, ×10^9^/L	11.8 (7.7, 17.9)	9.9 (6.5, 15.7)	11.8 (8.0, 16.8)	14.0 (9.3, 20.7)	<0.001
Platelets, ×10^9^/L	233.2 ± 125.2	242.5 ± 138.2	224.3 ± 121.1	232.1 ± 113.0	0.058
CK-MB,U/L	61.0 (38.0, 119.0)	48.0 (32.0, 81.0)	63.0 (41.0, 116.0)	79.0 (44.0, 171.5)	<0.001

hospital LOS, length of hospital stay; CHD, congenital heart disease; ICH, intracranial hemorrhage; AKI, acute renal injury; ALI, acute liver function injury; Total Ca, total calcium; ALT, alanine aminotransferase, BUN:blood urea nitrogen; Cr, creatinine; LAC, lactic acid; WBC, white blood cell count; CK-MB, creatine kinase-MB isoenzyme.

The median postnatal age of all enrolled neonates was 1 day (interquartile range: 0–7 days). Male neonates accounted for 61.2% (1,164/1,902), and preterm neonates accounted for 38.5% (731/1,902). The overall 28-day mortality and in-hospital death were 7.8% (148/1902) and 9.0% (172/1,902), respectively. The median hospital LOS was 20 days (IQR, 10.0–41.0 days).

Significant differences were observed among the Pi24 groups in several covariates, including postnatal age, 28-day mortality, in-hospital death, hospital LOS, congenital heart disease (CHD), neonatal asphyxia, sepsis, gastrointestinal malformations, acute kidney injury (AKI), serum phosphate, serum total calcium, alanine aminotransferase (ALT), blood urea nitrogen (BUN), creatinine (Cr), lactate (LAC), hemoglobin, white blood cell count (WBC), and creatine kinase-MB (CK-MB) levels (all *P* < 0.05) (Table 1).

### 3.3 Association between Serum phosphate levels and 28-day mortality

#### 3.3.1 Kaplan–Meier survival curve analysis of 28-day mortality Among critically Ill neonates

**Figure 2:**
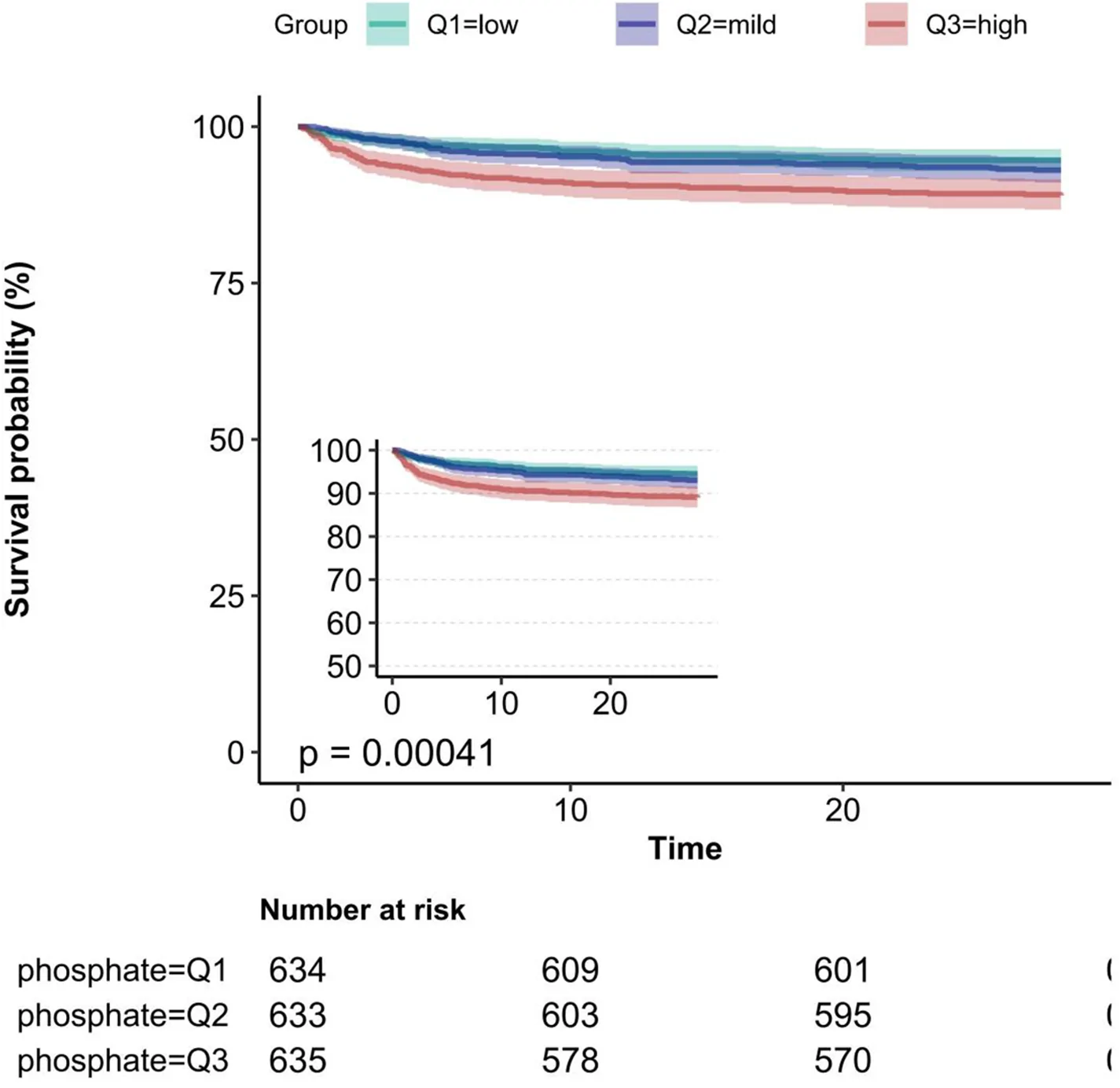
Kaplan–meier curve of 28-day mortality for critically ill newborns.

Survival analysis demonstrated significant differences in 28-day mortality among the three Pi24 groups. The Q3 group showed the highest 28-day mortality rate (11%) (log-rank *P* = 0.00041) (Figure 2).

#### 3.3.2 Multivariate Cox proportional hazards regression analysis

Univariate analysis showed that phosphate, premature, neonatal asphyxia, sepsis, ALI, AKI, prealbumin, ALT, BUN, Cr, LAC, WBC, platelet count, and CK-MB level were all associated with 28-day mortality (Supplementary Table S2).

Multivariate Cox proportional hazards regression analysis showed that when Pi24 was treated as a continuous variable, serum phosphate levels were associated with increased risk of 28-day mortality in unadjusted Model 1(HR,=1.29; 95% confidence interval [CI], 1.1–1.5; *P* = 0.001. However, this association was no longer statistically significant after adjustment for all confounding factors[HR,=1.15; 95% (CI), 0.94–1.41; *P* = 0.176]. When serum phosphate was analyzed as a tertiled categorical variable, the Q3 group exhibited a 76% higher mortality risk compared with the low serum phosphate group after full adjustment [HR,=1.76; 95% (CI), 1.08–2.88; *P* = 0.025] (Table 2).

**Table 2:** Multivariate Cox proprtional hazards regression analyses of phosphate level and 28-day mortality.

			Model I		Model Ⅱ		Model III		Model IV	
Variable	n.total	n.event_%	crude.HR (95%CI)	*P*-value	adj.HR (95%CI)	*P*-value	adj.HR (95%CI)	*P*-value	adj.HR (95%CI)	*P*-value
phosphate	1,902	148 (7.8)	1.29 (1.1∼1.5)	0.001	1.27 (1.08∼1.49)	0.003	1.29 (1.09∼1.52)	0.002	1.15 (0.94∼1.41)	0.176
Phosphate tertiles	1,902	148 (7.8)								
low	634	34 (5.4)	1(Ref)		1(Ref)		1(Ref)		1(Ref)	
mild	633	44 (7)	1.3 (0.83∼2.03)	0.251	1.29 (0.82∼2.03)	0.274	1.33 (0.85∼2.1)	0.212	1.2 (0.73∼2)	0.474
high	635	70 (11)	2.13 (1.42∼3.21)	<0.001	2.1 (1.37∼3.23)	0.001	2.1 (1.37∼3.21)	0.001	1.76 (1.08∼2.88)	0.025
Trend.test			1.48 (1.21∼1.82)	<0.001	1.47 (1.19∼1.82)	<0.001	1.46 (1.18∼1.81)	<0.001	1.34 (1.05∼1.72)	0.02

Model I: no other covariates were adjusted.

Model Ⅱ: we adjusted age, gender.

Model III：adjusted as for model Ⅱ,additionally adjusted for premature,Neonatal asphyxia,AKI, ALI.

ModelIV: adjusted as for model III,additionally adjusted for Laboratory examination (Prealbumin,WBC, Platelets, Ck-MB, LAC).

Smooth curve fitting was conducted to assess the potential nonlinear association between Pi24 and 28-day mortality; however, no significant correlation was identified (Supplementary Figure S1).

#### 3.3.3 Subgroup analysis and sensitivity analysis

**Figure 3:**
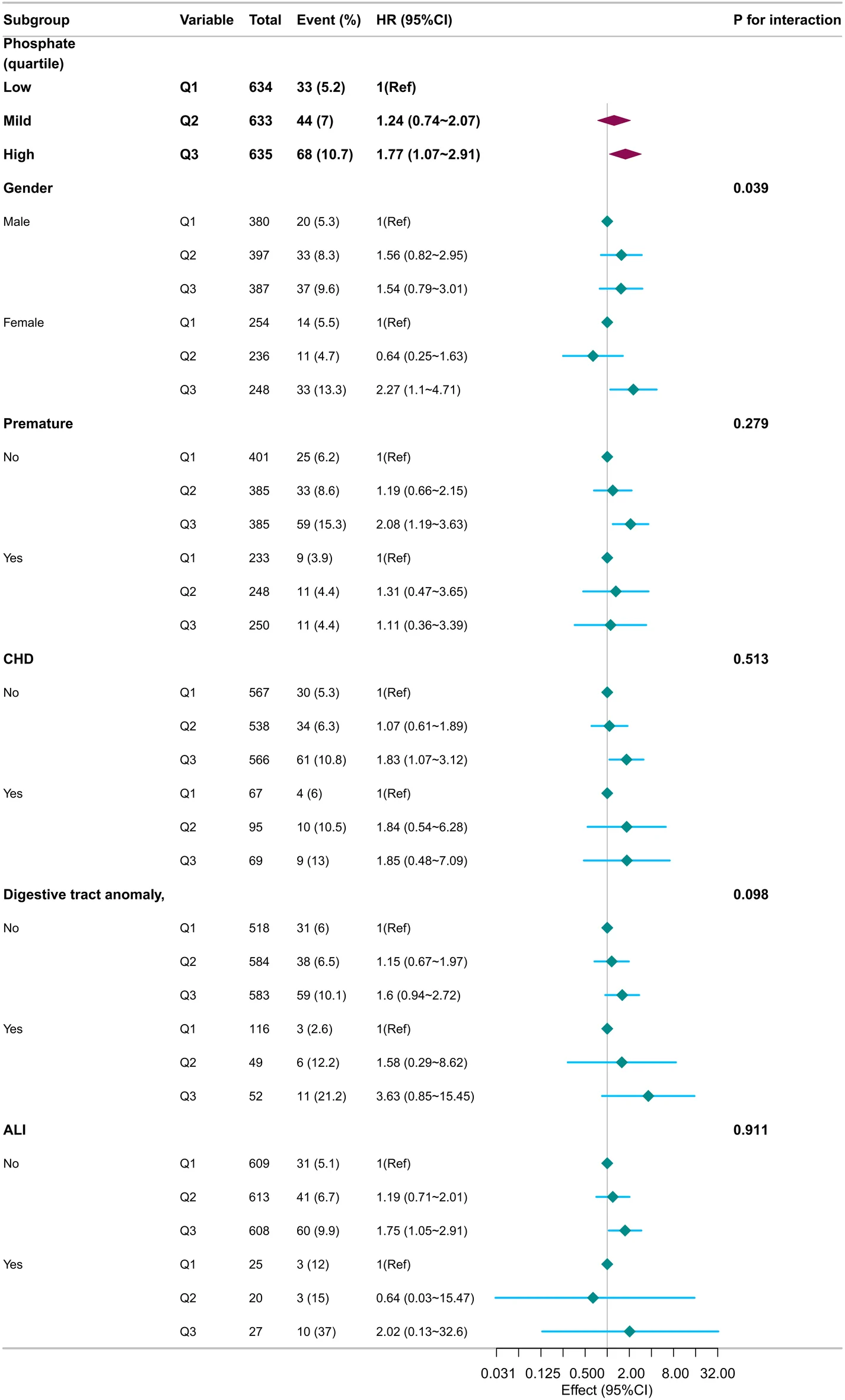
Subgroup analysis of the associations between Pi24 and 28-day mortality.

Subgroup analysis showed that higher Pi24 levels were significantly associated with increased mortality risk among female neonates, with a significant interaction effect (P for interaction = 0.039). No significant interactions were observed across subgroups stratified by preterm birth, CHD, gastrointestinal malformations, or ALI (Figure 3 and Supplementary Table S5).

Sensitivity analysis excluding neonates who died within 24 h after admission, those with congenital hereditary diseases, and those with tumors demonstrated consistent results. Compared with the Q1 group, neonates in the Q3 group had a 97% higher risk of 28-day mortality (HR = 1.97, 95% CI: 1.11–3.49, *P* = 0.021) (Supplementary Table S8). After additionally excluding neonates with AKI, the association remained similar but was no longer statistically significant (HR = 1.75, 95% CI: 0.97–3.15, *P* = 0.065) (Supplementary Table S9).

### 3.4 Association between serum phosphate levels and in-hospital death

#### 3.4.1 Multivariate logistic regression analysis

Univariate analysis showed that phosphate, hospital LOS, preterm birth, ALI, AKI, prealbumin, ALT, BUN, Cr, LAC, WBC, platelet count, and CK-MB level were all associated with in-hospital death (Supplementary Table S3).

Multivariate logistic regression analysis was applied to evaluate the independent association between serum phosphate levels and in-hospital death (Table 3). In the unadjusted Model I, higher serum phosphate levels were associated with an increased odds ratio (OR) for in-hospital death (OR = 1.27; 95% CI, 1.09–1.48; *P* = 0.003). However, after adjustment for all potential confounding factors (Model IV), continuous serum phosphate levels were not significantly associated with in-hospital death (OR = 1.19, 95% CI: 0.96–1.47, *P* = 0.113). When serum phosphate levels were analyzed as a categorical variable based on tertiles, neonates in the Q3 group had significantly higher odds of in-hospital death compared with those in the Q1 group (reference group) after full adjustment (OR = 1.80, 95% CI: 1.10–2.95, *P* = 0.019). This indicated that the odds of in-hospital death were approximately 80% higher in the Q3 group than in the Q1 group.

**Table 3:** Multivariate logistic regression analyses of phosphate level and in- hospital death.

			Model I		Model Ⅱ		Model III		Model IV	
Variable	n.total	n.event_%	crude.OR (95%CI)	*P*-value	adj.OR (95%CI)	*P*-value	adj.OR (95%CI)	*P*-value	adj.OR (95%CI)	*P*-value
phosphate	1,902	172 (9)	1.27 (1.09∼1.48)	0.003	1.25 (1.06∼1.47)	0.007	1.26 (1.07∼1.49)	0.007	1.19 (0.96∼1.47)	0.113
Phosphate tertiles	1,902	172 (9)								
Q1 Low	634	41 (6.5)	1(Ref)		1(Ref)		1(Ref)		1(Ref)	
Q2 mild	633	53 (8.4)	1.32 (0.87∼2.02)	0.197	1.3 (0.84∼1.99)	0.236	1.33 (0.86∼2.04)	0.2	1.27 (0.77∼2.09)	0.345
Q3 high	635	78 (12.3)	2.03 (1.36∼3.01)	<0.001	1.97 (1.3∼2.97)	0.001	1.96 (1.3∼2.97)	0.001	1.8 (1.1∼2.95)	0.019
Trend.test			1.43 (1.18∼1.75)	<0.001	1.41 (1.15∼1.74)	0.001	1.41 (1.15∼1.73)	0.001	1.35 (1.05∼1.72)	0.017

Model I: no other covariates were adjusted.

Model Ⅱ: we adjusted age, gender.

Model III: adjusted as for model Ⅱ,additionally adjusted for premature, Neonatal asphyxia, AKI, ALI.

ModelIV: adjusted as for model III,additionally adjusted for Laboratory examination（Prealbumin, WBC, Platelets, Ck-MB, LAC）.

**Figure 4:**
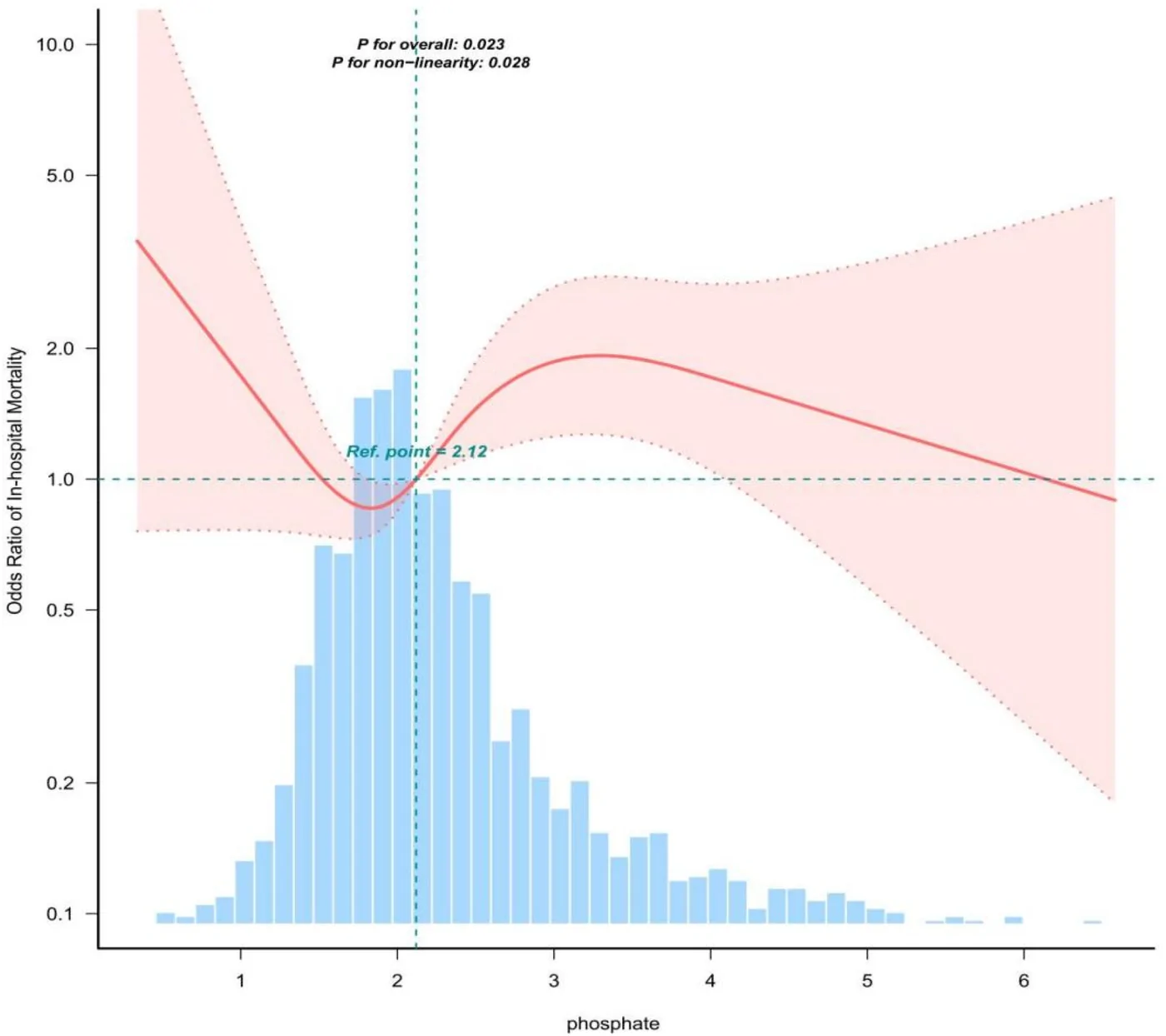
Nonlinear dose-response relationship between phosphate levels and in-hospital death. Adjustment factors included age,gender, premature, Neonatal asphyxia, ALI, AKI, Prealbumin, WBC, Platelets, Ck-MB, LAC. The red line and pink area represent the estimated values and their corresponding 95% confidence intervals, respectively.

Smooth curve fitting was performed to assess the potential nonlinear association between Pi24 and in-hospital death. After adjustment for confounding factors, a nonlinear relationship was observed between Pi24 and in-hospital death (Figure 4). Piecewise linear regression analysis identified two inflection points at serum phosphate levels of 1.76 mmol/L and 4.37 mmol/L. Within the range of 1.76–4.37 mmol/L, each 1.0 mmol/L increase in serum phosphate concentration was associated with a 45.6% increase in the odds of in-hospital death (OR = 1.456, 95% CI: 1.039–2.039, *P* = 0.029). No significant associations were observed when serum phosphate levels were <1.76 mmol/L (OR = 0.201, 95% CI: 0.036–1.115, *P* = 0.066) or ≥4.37 mmol/L (OR = 0.017, 95% CI: 0.000–13.57, *P* = 0.231) (Table 4).

**Figure 5:**
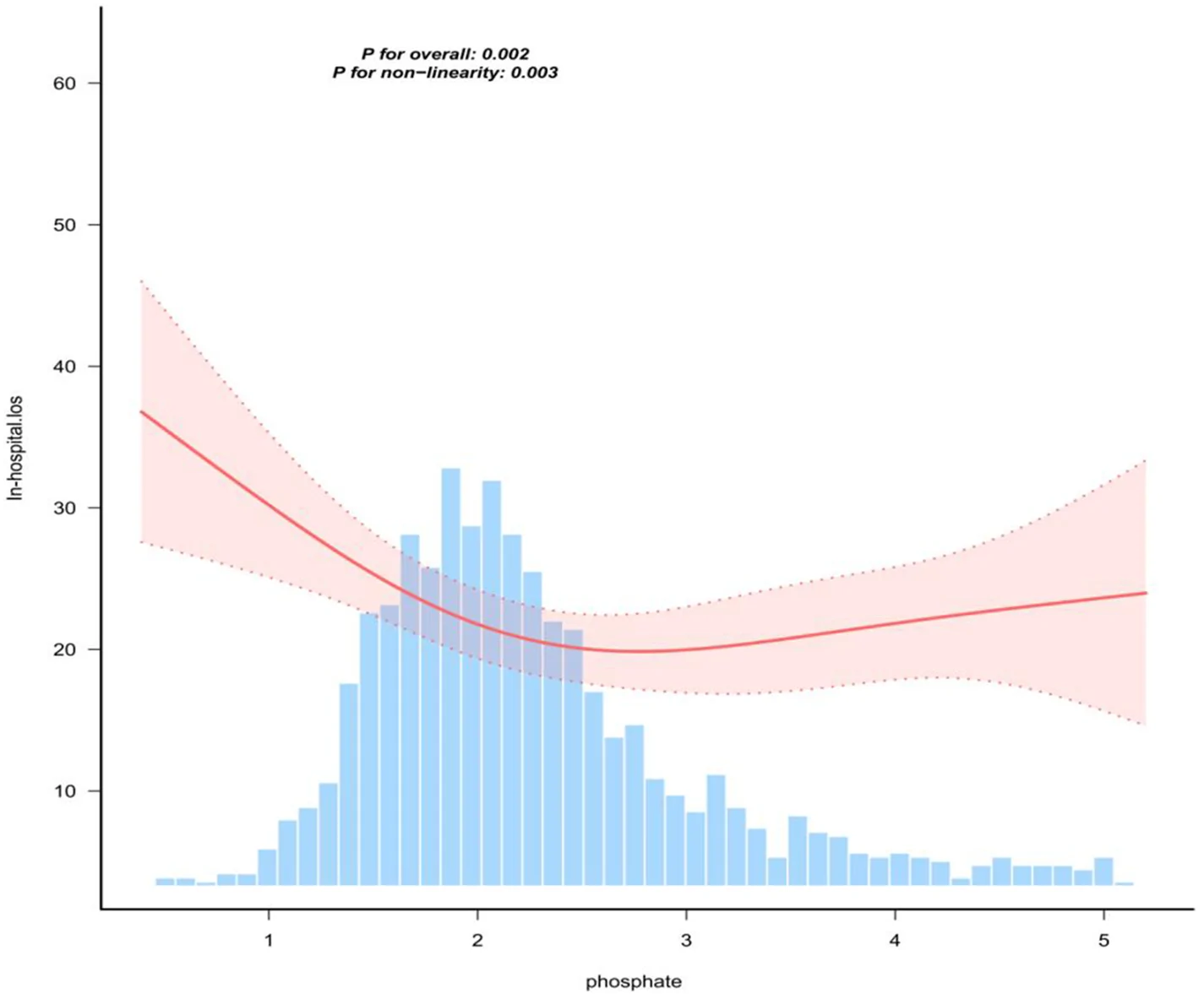
Nonlinear dose-response relationship between phosphate level and hospital LOS. Adjustment factors included age, gender, premature, Neonatal asphyxia, Sepsis and Digestive tract anomaly, Hemoglobin, WBC, Platelet, Ck-MB, BUN, Prealbumin, LAC.

**Table 4:** Threshold effect analysis of phosphate levels on in-hospital death.

Threshold of serm phosphate(mmol/L)	Adjusted OR (95% CI)	*P*-value
phosphate<1.76	0.201 (0.036∼1.115)	0.066
phosphate≥1.76,<4.37	1.456 (1.039∼2.039)	0.029
phosphate≥4.37	0.017 (0∼13.57)	0.2313
Likelihood Ratio test	-	0.014

Adjustment factors included age, gender, premature, Neonatal asphyxia, ALI, AKI, Prealbumin, WBC, Platelets, Ck-MB, LAC.

#### 3.4.2 Subgroup analysis and sensitivity analysis

Subgroup analysis showed no significant interaction between Pi24 and in-hospital death across subgroups stratified by sex, preterm birth, CHD, and ALI (all P for interaction >0.05) (Supplementary Figure S2 and Table S6).

Sensitivity analysis excluding neonates who died within 24 h after admission, those with congenital hereditary diseases, and those with tumors showed consistent results. Compared with the Q1 group, neonates in the Q3 group had significantly higher odds of in-hospital death (OR = 1.91, 95% CI: 1.12–3.28, *P* = 0.018) (Supplementary Table S10). After additionally excluding neonates with AKI, the association remained similar but was no longer statistically significant (OR = 1.71, 95% CI: 0.98–2.98, *P* = 0.057) (Supplementary Table S11).

### 3.5 Association between serum phosphate levels and hospital LOS

#### 3.5.1 Multivariate linear regression analysis

After excluding 172 neonates who died during hospitalization, the association between Pi24 and hospital LOS was evaluated among 1,730 surviving neonates. Univariate analysis showed that phosphate levels, postnatal age, preterm birth, neonatal asphyxia, sepsis, digestive tract anomaly, prealbumin, BUN, LAC, hemoglobin, WBC, and platelet count were significantly associated with hospital LOS (Supplementary Table S4).

Multivariate linear regression analysis was performed to evaluate the association between Pi24 and hospital LOS (Table 5). When Pi24 was analyzed as a continuous variable, no significant association was observed between serum phosphate levels and hospital LOS after full adjustment for confounding factors (Model IV) (*β* = −1.33, 95% CI: −2.86 ∼ 0.21, *P* = 0.090).

**Table 5:** Multi-variable linear regression analyses of phosphate level and in- hospital LOS.

		Model I		Model Ⅱ		Model III		Model IV	
Variable	n.total	crude.*β* (95%CI)	*P* value	adj.β (95%CI)	*P*-value	adj.β(95%CI)	*P*-value	adj.β(95%CI)	*P*-value
phosphate	1,730	−1.06 (−2.4∼0.28)	0.12	−0.34 (−1.73∼1.06)	0.636	−1 (−2.25∼0.26)	0.12	−1.33 (−2.86∼0.21)	0.09
Phosphate tertiles	1,730								
Q1 Low	562	0(Ref)		0(Ref)		0(Ref)		0(Ref)	
Q2 mild	590	−5.05 (−7.99∼-2.12)	0.001	-4.17 (−7.16∼-1.19)	0.006	-5.18 (−7.89∼-2.48)	<0.001	-4.54 (−7.46∼-1.62)	0.002
Q3 high	578	-4.35 (−7.3∼-1.4)	0.004	-2.97 (−6.05∼0.11)	0.059	-4.68 (−7.47∼-1.89)	0.001	-4.68 (−7.84∼-1.53)	0.004
Trend.test		-2.16 (−3.64∼-0.68)	0.004	-1.45 (−2.99∼0.09)	0.066	-2.27 (−3.66∼-0.87)	0.001	-2.35 (−3.92∼-0.77)	0.004

Model I: no other covariates were adjusted.

Model Ⅱ: we adjusted age, gender.

Model III: adjusted as for model Ⅱ,additionally adjusted for Premature, Neonatal asphyxia, Sepsis and Digestive tract anomaly.

ModelIV: adjusted as for model III,additionally adjusted for Laboratory examination（Hemoglobin, WBC, Platelet, Ck-MB, BUN, Prealbumin, LAC）.

When Pi24 was analyzed as a categorical variable according to tertiles, neonates in the Q1 group had a significantly longer hospital LOS compared with those in the Q3 group, with an average prolongation of 4.68 days (*β* = −4.68, 95% CI: −7.84 ∼ −1.53, *P* = 0.004).

Smooth curve fitting was performed to evaluate the nonlinear association between Pi24 and hospital LOS. After adjustment for confounding factors, an L-shaped relationship was observed between Pi24 and hospital LOS (Figure 5). Threshold effect analysis identified a single inflection point at a serum phosphate concentration of 2.5 mmol/L. When serum phosphate levels were <2.5 mmol/L, each 1.0 mmol/L decrease in serum phosphate concentration was associated with a 6.89-day prolongation in hospital LOS (*β* = −6.892, 95% CI: −10.801 ∼−2.983, *P* = 0.001). However, when serum phosphate levels were ≥2.5 mmol/L, no significant association was observed between serum phosphate levels and hospital LOS (*β* = 1.995, 95% CI: −1.136 ∼ 5.125, *P* = 0.211) (Table 6).

**Table 6:** Threshold effect analysis of phosphate levels on in-hospital LOS.

Threshold of serm phosphate(mmol/L)	Adjusted β (95% CI)	*P*-value
phosphate<2.5	−6.892 (−10.801∼-2.983)	0.001
phosphate≥2.5	1.995 (−1.136∼5.125)	0.211
Likelihood Ratio test	-	<0.001

Adjustment factors included age,gender, premature,neonatal asphyxia,sepsis and digestive tract anomaly, hemoglobin, WBC, platelet, Ck-MB, BUN, prealbumin, LAC.

#### 3.5.2 Subgroup analysis and sensitivity analysis

Subgroup analysis revealed no significant interaction between Pi24 and hospital LOS across subgroups stratified by gender, preterm birth, CHD, AKI and ALI (all P-interaction > 0.05) (Supplementary Figure S3 and Table S7). Sensitivity analysis excluding neonates who died within the first 24 h after admission, those with congenital hereditary diseases, those with tumour and AKI ramains stable (*β*=-4.12; 95% CI, −7.34∼-0.9; *P* = 0.012)(Supplementary Table S12).

## 4 Discussion

Studies investigating the association between serum phosphate levels and clinical outcomes in critically ill neonates remain limited. The major findings of the present study were that elevated Pi24 was associated with increased risks of 28-day mortality and in-hospital death odds, and that Pi24 exhibited nonlinear associations with in-hospital death and hospital LOS after adjustment for potential confounding factors. Within the serum phosphate range of 1.76–4.37 mmol/L, each 1.0 mmol/L increase in serum phosphate concentration was associated with a 45.6% increase in the odds of in-hospital death. Furthermore, hypophosphatemia was independently associated with prolonged hospital LOS; when serum phosphate levels were below 2.5 mmol/L, each 1.0 mmol/L decrease in phosphate concentration was associated with a 6.89-day prolongation of hospital LOS.

Critically ill patients are vulnerable to disturbances in electrolyte and mineral metabolism, including alterations in serum phosphate levels (12). However, the definitions of abnormal serum phosphate levels and their prognostic implications vary across different populations. In adults, hypophosphatemia is defined as a serum phosphate concentration <0.8 mmol/L (2.5 mg/dL) (13), whereas hyperphosphatemia is defined as a serum phosphate concentration ≥1.45 mmol/L (4.5 mg/dL) (14). Neonates require relatively higher physiological phosphate levels to support rapid growth and development. Serum phosphate levels in neonates undergo dynamic changes, with concentrations being relatively low at birth, increasing during the first 48 h of life, and subsequently declining as part of normal physiological maturation. These changes are influenced by postnatal age, feeding regimen, hormonal regulation, and renal functional maturation (15).

Given that reference intervals for serum phosphate vary across different postnatal ages, we categorized Pi24 into three groups to evaluate its associations with clinical outcomes. Meanwhile, to assess the prevalence of hypophosphatemia and hyperphosphatemia, baseline characteristics were additionally stratified according to clinically relevant cut-off values (1.6 mmol/L and 2.6 mmol/L). The reference range for normal serum phosphate levels (1.6–2.6 mmol/L) was determined based on the reference interval of our hospital clinical laboratory and previously published studies (8, 9, 16, 17).

The prevalence of phosphate metabolism disorders varies considerably among critically ill populations. Hypophosphatemia occurs in approximately 30% of adult ICU patients, while the reported incidence of hyperphosphatemia ranges from 5.6% to 45% among critically ill adults (14). In pediatric ICU patients, the prevalence of hypophosphatemia at admission is approximately 42%, and hyperphosphatemia has been reported in 22.2% of patients (18, 19). However, data regarding phosphate metabolism disturbances in critically ill neonates remain limited. Previous studies have reported hypophosphatemia rates ranging from 60% to 80% among neonates with sepsis (20, 21), while hyperphosphatemia has been reported in 15.6%–26% of neonates (9, 21). Among preterm infants with refeeding syndrome, the incidence of hypophosphatemia can reach 77% (8).

In the present study, the admission prevalence of hypophosphatemia (<1.6 mmol/L) was 15.7%, whereas hyperphosphatemia (>2.6 mmol/L) was observed in 28.5% of neonates (SupplementaryTable S1). The differences in the reported prevalence of phosphate metabolism disorders across studies may be attributable to variations in diagnostic criteria, underlying disease characteristics, and timing of serum phosphate measurements.

Baseline comparisons among different phosphate groups revealed significant differences in postnatal age. Neonates in the hypophosphatemia group had the highest postnatal age, which was consistent with the physiological pattern of declining serum phosphate levels with increasing postnatal age (4). Neonates with hypophosphatemia also exhibited a longer hospital LOS and higher proportions of sepsis and gastrointestinal malformations, consistent with previous reports (21, 22). In contrast, the hyperphosphatemia group showed higher proportions of neonatal asphyxia, AKI, and elevated CK-MB and lactate levels. These findings may be explained by the close association between hyperphosphatemia and critical illness-related conditions, including tissue ischemia, hypoxia, cellular apoptosis, tissue injury, and impaired renal function (23–25).

The association between phosphate metabolism disorders and mortality has been extensively investigated in adult and pediatric populations; however, the conclusions remain inconsistent. Most previous studies have identified hyperphosphatemia as an independent risk factor for mortality and adverse clinical outcomes (3, 19, 26–28). Regarding hypophosphatemia, several studies have demonstrated its predictive value for poor clinical outcomes (28–30), whereas others have failed to identify a significant association (3, 31). In neonates, some studies have reported that hypophosphatemia during the first 5 days after birth is associated with increased mortality among preterm infants (32, 33), while other studies have not confirmed this relationship (8, 34). Therefore, the prognostic significance of hypophosphatemia in neonatal mortality remains uncertain. Furthermore, existing studies examining hypophosphatemia and neonatal prognosis have mainly focused on preterm infants with refeeding syndrome, with relatively small sample sizes, limited adjustment for potential confounders, and insufficient evaluation of nonlinear or threshold relationships between phosphate levels and clinical outcomes. In the present study, higher Pi24 was independently associated with increased risks of 28-day mortality and in-hospital mortality in critically ill neonates, and subgroup analyses further supported the robustness of these findings (Tables 3, 4).

Smooth curve fitting demonstrated a nonlinear association between serum phosphate levels and in-hospital mortality after full adjustment for potential confounding factors, which was consistent with previous findings in pediatric ICU populations (28). Threshold effect analysis identified two inflection points at serum phosphate concentrations of 1.76 mmol/L and 4.37 mmol/L. When serum phosphate levels were below 1.76 mmol/L, lower phosphate levels showed a tendency toward increased mortality risk, although the association did not reach statistical significance (*P* = 0.066). Within the range of 1.76–4.37 mmol/L, each 1.0 mmol/L increase in serum phosphate concentration was associated with a 45.6% increase in the odds of in-hospital mortality. When serum phosphate levels exceeded 4.37 mmol/L, the curve reached a plateau, suggesting a possible saturation effect. However, this finding should be interpreted cautiously, as the number of neonates with extremely elevated phosphate levels was limited, resulting in unstable estimates and a wide confidence interval (95% CI: 0–13.57). Collectively, these findings suggest that hyperphosphatemia is closely associated with increased in-hospital death, whereas the prognostic implications of lower phosphate levels and extremely elevated phosphate levels require further investigation.

The present study further characterized the threshold relationship between admission serum phosphate levels and odds of in-hospital death in critically ill neonates, which differed from previously reported threshold patterns in critically ill adults and general PICU populations. Guo et al. reported a nonlinear association between serum phosphate levels and 30-day all-cause mortality in adult patients with sepsis, showing that mortality risk increased with phosphate levels below 6.8 mg/dL, whereas no further association was observed above this threshold (35). Similarly, a PICU cohort study conducted by Wang et al. demonstrated an inverse association between phosphate levels and 30-day mortality below 1.2 mmol/L, but a positive association above this threshold (28). The differences in threshold values and curve patterns among studies may be attributed to variations in patient populations, disease characteristics, and underlying pathophysiological conditions.

To minimize the potential confounding effect of in-hospital mortality on hospital LOS, we further evaluated the association between Pi24 and hospital LOS after excluding neonates who died during hospitalization. The results demonstrated that lower Pi24 was significantly associated with prolonged hospital LOS, consistent with previous studies (21, 30, 34, 36). Importantly, we identified an L-shaped association between Pi24 and hospital LOS in critically ill neonates. When serum phosphate levels were <2.5 mmol/L, each 1.0 mmol/L decrease in phosphate concentration was associated with a 6.89-day prolongation of hospital LOS, whereas phosphate fluctuations above this threshold were not significantly associated with hospital LOS. A previous PICU study reported prolonged hospitalization among children younger than 2 years undergoing cardiac surgery with serum phosphate levels <1.0 mmol/L; however, that study relied on categorical comparisons rather than smooth curve fitting and threshold effect analyses to identify clinically relevant cut-off values (34).

Phosphorus homeostasis is essential for maintaining cellular integrity, muscle contraction, nerve conduction, and multiple organ functions, and therefore may play an important role in clinical outcomes. Previous studies have demonstrated that young mammals exhibit enhanced intestinal phosphate absorption and renal tubular phosphate reabsorption compared with adults, supporting rapid skeletal mineralization and maintaining positive phosphate balance. Systemic phosphate homeostasis is regulated by multiple hormones, including parathyroid hormone (PTH), fibroblast growth factor 23 (FGF23), calcitriol, growth hormone (GH), triiodothyronine (T3), and glucocorticoids, through modulation of intestinal absorption and renal phosphate handling (4).

Hyperphosphatemia in critically ill patients is primarily attributed to impaired renal phosphate excretion caused by kidney dysfunction and extracellular phosphate release secondary to cellular injury under various critical conditions (23–25). Since renal function is physiologically immature in neonates, their ability to compensate for disturbances in phosphate metabolism may be substantially limited (37). Conversely, hypophosphatemia in critically ill patients commonly results from intracellular phosphate redistribution and renal phosphate loss, which may occur in conditions such as sepsis, postoperative states, trauma, aggressive fluid resuscitation, refeeding syndrome, acid–base disturbances, medication exposure (including glucose/insulin, catecholamines, and diuretics), and renal replacement therapy (17, 38, 39). FGF23 is highly responsive to hypoxemia and inflammatory stimuli, and its upregulation may contribute to reduced serum phosphate levels in critically ill neonates (40). Acute hypophosphatemia may impair multiple organ systems, including respiratory function, myocardial contractility, cardiac rhythm, skeletal muscle integrity, erythrocyte function, leukocyte activity, and insulin sensitivity (41). Therefore, phosphate depletion in critically ill patients may delay clinical recovery and contribute to prolonged hospitalization.

Sensitivity analyses excluding neonates who died within 24 h after admission, those with congenital hereditary diseases, or those with tumors demonstrated consistent results. However, after additionally excluding neonates with AKI, Pi24 was no longer significantly associated with 28-day mortality (*P* = 0.065) or in-hospital mortality (*P* = 0.057), whereas the association between Pi24 and hospital LOS remained statistically significant. Two potential explanations may account for this finding. First, the kidney plays a central role in maintaining serum phosphate homeostasis; therefore, AKI may substantially influence the relationship between hyperphosphatemia and mortality risk. Second, the relatively small number of mortality events may have reduced the statistical power and robustness of the mortality analyses.

Several limitations of this study should be acknowledged. First, this was a single-center retrospective cohort study, and selection bias and residual confounding could not be completely eliminated despite adjustment for major confounding factors and the performance of subgroup and sensitivity analyses. Second, serum phosphate levels were assessed only within 24 h of admission, and subsequent dynamic changes in phosphate concentrations during NICU hospitalization were not available, which may have limited the evaluation of long-term prognostic implications. Third, detailed medication information in the PIC database was incomplete; therefore, we were unable to further investigate whether phosphate supplementation or fluid management strategies influenced clinical outcomes. Fourth, the diagnosis of AKI in this study was based solely on serum creatinine criteria corresponding to stage 1–3 AKI according to the neonatal KDIGO classification system, without incorporating urine output criteria. This limitation may have affected the accuracy of AKI identification and introduced potential residual confounding. Fifth, the relatively small number of mortality events may have limited the statistical power and reduced the robustness of the observed associations.

Future multicenter prospective studies with larger sample sizes are warranted to validate the prognostic significance of dynamic changes in serum phosphate levels and further clarify the potential impact of phosphate supplementation on clinical outcomes in critically ill neonates.

## 5 Conclusion

Higher Pi24 was associated with increased risks of 28-day mortality and in-hospital death odds among critically ill neonates. Pi24 demonstrated nonlinear associations with clinical outcomes: Pi24 within the range of 1.76–4.37 mmol/L were associated with increased odds of in-hospital mortality, whereas lower Pi24 (<2.5 mmol/L) were associated with prolonged hospital length of stay. These findings suggest that critically ill neonates may be particularly vulnerable to disturbances in phosphate homeostasis, and early monitoring of serum phosphate levels may help identify neonates at higher risk of adverse outcomes. Further prospective studies are needed to determine whether targeted management of phosphate levels can improve clinical outcomes in this population.

## Data Availability

The datasets presented in this study can be found in online repositories. The names of the repository/repositories and accession number(s) can be found in the article/Supplementary Material.
