# Supplementary material for: Association between serum phosphate levels and outcome among critically ill newborns: a retrospective cohort study
Source: Front Pediatr. 2026 Aug 27;14:1909660. doi: 10.3389/fped.2026.1909660 (PMC13558138; doi:10.3389/fped.2026.1909660)
Supplement: Supplementary file 1 [file Supplementaryfile1.docx]

Figure S1 Curve fitting plot demonstrating the association between serum phosphate levels and 28-day mortality. Adjustment factors included age,gender,premature, Neonatal asphyxia, ALI, AKI, Prealbumin, WBC, Platelets, Ck-MB, LAC. The red line and pink area represent the estimated values and their corresponding 95% confidence intervals, respectively.

Figure S2 :Subgroup analysis of the associations between phosphate levels and in-hospital death

Figure S3 Subgroup analysis of the associations between phosphate levels and hospital LOS

**Table S1 Baseline characteristics of participants**

| **Co variate** | **Total** | **Phosphate level(mmol/L)** | | | **P-value** |
| --- | --- | --- | --- | --- | --- |
|  |  | Low(<1.6) | Mild(1.6-2.6) | High(>2.6) |  |
|  | (n = 1902) | (n = 299) | (n = 1060) | (n =543) |  |
| **Baseline characteristics** |  |  |  |  |  |
| gender, males n (%) | 1164 (61.2) | 182 (60.9) | 656 (61.9) | 326 (60) | 0.766 |
| age,days | 1.0 (0.0, 7.0) | 6.0 (1.0, 15.5) | 1.0 (0.0, 8.0) | 1.0 (0.0, 2.0) | < 0.001 |
| premature, n (%) | 731 (38.5) | 127 (42.5) | 386 (36.4) | 218 (40.1) | 0.102 |
| **Outcome** |  |  |  |  |  |
| 28-day Mortality, n(%) | 148 ( 7.8) | 18 (6) | 67 (6.3) | 63 (11.6) | < 0.001 |
| In-hospital death, n (%) | 172 ( 9.0) | 20 (6.7) | 83 (7.8) | 69 (12.7) | 0.002 |
| hospital.Los, days | 20.0 (10.0, 41.0) | 27.0 (13.0, 48.0) | 20.0 (10.0, 39.0) | 16.0 (8.0, 39.0) | < 0.001 |
| **Comorbidities** |  |  |  |  |  |
| CHD, n (%) | 231 (12.1) | 31 (10.4) | 139 (13.1) | 61 (11.2) | 0.326 |
| Neonatal asphyxia, n (%) | 201 (10.6) | 20 (6.7) | 102 (9.6) | 79 (14.5) | < 0.001 |
| Sepsis, n (%) | 82 ( 4.3) | 21 (7) | 40 (3.8) | 21 (3.9) | 0.042 |
| Digestive tract anomaly, n (%) | 217 (11.4) | 58 (19.4) | 117 (11) | 42 (7.7) | < 0.001 |
| ICH, n (%) | 51 ( 2.7) | 7 (2.3) | 30 (2.8) | 14 (2.6) | 0.888 |
| ALI, n (%) | 72 ( 3.8) | 9 (3) | 41 (3.9) | 22 (4.1) | 0.734 |
| AKI, n (%) | 59 ( 3.1) | 6 (2) | 26 (2.5) | 27 (5) | 0.011 |
| **Laboratory examination** |  |  |  |  |  |
| phosphate, mmol/L | 2.4 ± 0.9 | 1.3 ± 0.2 | 2.1 ± 0.3 | 3.5 ± 0.8 | < 0.001 |
| TotalCa,mmol/L | 2.1 ± 0.3 | 2.2 ± 0.3 | 2.1 ± 0.3 | 2.1 ± 0.4 | 0.001 |
| Prealbumin, g/L | 0.1 ± 0.0 | 0.1 ± 0.0 | 0.1 ± 0.0 | 0.1 ± 0.0 | 0.229 |
| ALT,U/L | 11.0 (7.0, 18.0) | 12.0 (6.0, 21.0) | 10.0 (6.0, 17.0) | 11.0 (7.0, 18.0) | 0.014 |
| BUN, mmol/L | 5.1 ± 3.2 | 4.9 ± 3.3 | 4.8 ± 3.1 | 5.8 ± 3.2 | < 0.001 |
| Cr, µmol/L | 80.4 ± 34.5 | 63.2 ± 28.8 | 78.0 ± 35.1 | 94.8 ± 30.5 | < 0.001 |
| LAC,mmol/L | 2.6 (1.7, 4.1) | 1.8 (1.3, 2.9) | 2.6 (1.7, 4.0) | 3.0 (2.0, 4.8) | < 0.001 |
| Hemoglobin,g/L | 146.6 ± 37.7 | 127.2 ± 33.6 | 145.9 ± 36.6 | 158.7 ± 37.4 | < 0.001 |
| WBC, ×10^9^/L | 11.8 (7.7, 17.9) | 9.4 (6.3, 15.3) | 11.5 (7.6, 17.0) | 14.2 (9.2, 20.9) | < 0.001 |
| Platelets, ×10^9^/L | 233.2 ± 125.2 | 240.0 ± 148.0 | 231.6 ± 122.8 | 232.2 ± 114.6 | 0.626 |
| Ck-MB,U/L | 61.0 (38.0, 119.0) | 41.0 (29.0, 64.0) | 61.0 (40.0, 111.8) | 79.0 (44.0, 169.0) | < 0.001 |

Abbreviations: hospital.Los.:length of hospital stay, CHD: congenital heart disease; ICH: intracranial hemorrhage; AKI:acute renal injury; ALI:acute liver function injury, Total Ca, total calcium;ALT: alanine aminotransferase, BUN: blood urea nitrogen, Cr:creatinine, LAC:lactic acid, WBC:white blood cell count, CK-MB:creatine kinase-MB isoenzyme.

**Table S2 Association of covariates and 28-day Mortality**

| Variable | HR_95CI | P_value |
| --- | --- | --- |
| gender | 0.99(0.71~1.39 | 0.977 |
| age | 0.98 (0.96~1.01) | 0.157 |
| premature | 0.39 (0.26,0.58) | <0.001 |
| CHD | 1.34 (0.86,2.1) | 0.208 |
| Neonatal asphyxia | 1.77 (1.14,2.74) | 0.017 |
| Sepsis | 1.32 (0.65,2.7) | 0.459 |
| ICH | 0.24 (0.03,1.74) | 0.068 |
| Digestive tract anomaly | 1.25 (0.78,2.01) | 0.359 |
| ALI | 3.34 (1.96,5.7) | <0.001 |
| AKI | 2.79 (1.51,5.17) | 0.004 |
| Phosphate | 1.28 (1.1,1.5) | 0.003 |
| Prealbumin | 0 (0,0.04) | 0.002 |
| ALT | 1 (1~1) | <0.001 |
| BUN | 1.06 (1.03,1.1) | 0.002 |
| CR | 1.01 (1~1.01) | <0.001 |
| Lac | 1.17 (1.14,1.2) | <0.001 |
| TotalCa | 1.08 (0.64,1.82) | 0.774 |
| Hemoglobin | 1 (0.99~1) | 0.262 |
| WBC | 1.01 (1,1.03) | 0.034 |
| Platelets | 1 (0.99~1) | <0.001 |
| Ck-MB | 1 (1~1) | <0.001 |

**Table S3 Association of covariates and in-hospital death**

| Variable | OR_95CI | P_value |
| --- | --- | --- |
| gender | 0.98 (0.71~1.35) | 0.904 |
| age | 0.98 (0.96~1.01) | 0.15 |
| hospital.los | 0.95 (0.94~0.96) | <0.001 |
| premature | 0.54 (0.38~0.77) | 0.001 |
| CHD | 1.32 (0.85~2.06) | 0.212 |
| Neonatal asphyxia | 1.5 (0.96~2.36) | 0.078 |
| Sepsis | 1.25 (0.62~2.55) | 0.534 |
| ICH | 0.2 (0.03~1.43) | 0.108 |
| Digestive tract anomaly | 1.22 (0.77~1.94) | 0.397 |
| ALI | 3.34 (1.89~5.9) | <0.001 |
| AKI | 2.39 (1.22~4.7) | 0.011 |
| Phosphate | 1.27 (1.09~1.48) | 0.003 |
| Prealbumin | 0 (0~0.08) | 0.005 |
| ALT | 1 (1~1) | 0.001 |
| BUN | 1.07 (1.03~1.11) | 0.001 |
| CR | 1.01 (1~1.01) | <0.001 |
| Lac | 1.18 (1.14~1.22) | <0.001 |
| TotalCa | 0.96 (0.58~1.58) | 0.874 |
| Hemoglobin | 1 (0.99~1) | 0.322 |
| WBC | 1.02 (1~1.03) | 0.008 |
| Platelets | 1 (0.99~1) | <0.001 |
| Ck-MB | 1 (1~1) | <0.001 |

**Table S4 Association of covariates and hospital LOS.**

| Variable | Coeff.(95%CI) | P |
| --- | --- | --- |
| gender | 0.15 (-2.32,2.62) | 0.905 |
| age | 0.3 (0.14,0.45) | < 0.001 |
| premature | 23.45 (21.25,25.64) | < 0.001 |
| CHD | -1.09 (-4.81,2.63) | 0.567 |
| Neonatal asphyxia | -14.74 (-18.66,-10.83) | < 0.001 |
| Sepsis | -6.79 (-12.76,-0.81) | 0.026 |
| ICH | -2.59 (-9.77,4.58) | 0.479 |
| Digestive tract anomaly | -8.89 (-12.68,-5.11) | < 0.001 |
| ALI | 3.54 (-3.32,10.39) | 0.311 |
| AKI | 3.18 (-4.14,10.5) | 0.395 |
| phosphate | -1.06 (-2.4,0.28) | 0.12 |
| Hemoglobin | -0.08 (-0.11,-0.04) | < 0.001 |
| WBC | -0.19 (-0.32,-0.07) | 0.003 |
| Platelets | -0.02 (-0.03,-0.01) | < 0.001 |
| CK-MB | 0 (-0.01,0) | 0.774 |
| ALT | -0.02 (-0.03,0) | 0.07 |
| BUN | 1.29 (0.91,1.66) | < 0.001 |
| CR | -0.02 (-0.05,0.02) | 0.303 |
| PH | -12.01 (-23.75,-0.27) | 0.045 |
| Lac | -1.16 (-1.58,-0.74) | < 0.001 |
| TotalCa | -1.01 (-4.94,2.92) | 0.613 |
| Prealbumin | -61.8 (-103.01,-20.6) | 0.003 |

**Table S5 Subgroup analysis of the associations between phosphate levels and 28-day mortality**

| **Subgroup** | **Variable** | **n.total** | **n.event_%** | **crude.HR_95CI** | **crude.P_value** | **adj.HR_95CI** | **adj.P_value** | **P.for.interaction_1** |
| --- | --- | --- | --- | --- | --- | --- | --- | --- |
| **Gender** |  |  |  |  |  |  |  | 0.039 |
| Male | Q1 | 380 | 20 (5.3) | 1(Ref) |  | 1(Ref) |  |  |
|  | Q2 | 397 | 33 (8.3) | 1.59 (0.91~2.77) | 0.101 | 1.56 (0.82~2.95) | 0.172 |  |
|  | Q3 | 387 | 37 (9.6) | 1.88 (1.09~3.24) | 0.023 | 1.54 (0.79~3.01) | 0.201 |  |
|  | Trend.test | 1164 | 90 (7.7) | 1.35 (1.04~1.75) | 0.024 | 1.21 (0.88~1.66) | 0.23 |  |
| Female | Q1 | 254 | 14 (5.5) | 1(Ref) |  | 1(Ref) |  |  |
|  | Q2 | 236 | 11 (4.7) | 0.84 (0.38~1.85) | 0.667 | 0.64 (0.25~1.63) | 0.346 |  |
|  | Q3 | 248 | 33 (13.3) | 2.51 (1.34~4.69) | 0.004 | 2.27 (1.1~4.71) | 0.027 |  |
|  | Trend.test | 738 | 58 (7.9) | 1.71 (1.23~2.39) | 0.002 | 1.63 (1.1~2.43) | 0.015 |  |
| **Premature** |  |  |  |  |  |  |  | 0.279 |
| No | Q1 | 401 | 25 (6.2) | 1(Ref) |  | 1(Ref) |  |  |
|  | Q2 | 385 | 33 (8.6) | 1.38 (0.82~2.33) | 0.22 | 1.19 (0.66~2.15) | 0.572 |  |
|  | Q3 | 385 | 59 (15.3) | 2.6 (1.63~4.16) | <0.001 | 2.08 (1.19~3.63) | 0.01 |  |
|  | Trend.test | 1171 | 117 (10) | 1.65 (1.31~2.08) | <0.001 | 1.48 (1.12~1.95) | 0.007 |  |
| Yes | Q1 | 233 | 9 (3.9) | 1(Ref) |  | 1(Ref) |  |  |
|  | Q2 | 248 | 11 (4.4) | 1.14 (0.47~2.76) | 0.766 | 1.31 (0.47~3.65) | 0.602 |  |
|  | Q3 | 250 | 11 (4.4) | 1.14 (0.47~2.76) | 0.765 | 1.11 (0.36~3.39) | 0.859 |  |
|  | Trend.test | 731 | 31 (4.2) | 1.07 (0.69~1.65) | 0.77 | 1.04 (0.61~1.79) | 0.882 |  |
| **CHD** |  |  |  |  |  |  |  | 0.513 |
| No | Q1 | 567 | 30 (5.3) | 1(Ref) |  | 1(Ref) |  |  |
|  | Q2 | 538 | 34 (6.3) | 1.2 (0.73~1.95) | 0.475 | 1.07 (0.61~1.89) | 0.815 |  |
|  | Q3 | 566 | 61 (10.8) | 2.11 (1.36~3.26) | 0.001 | 1.83 (1.07~3.12) | 0.027 |  |
|  | Trend.test | 1671 | 125 (7.5) | 1.48 (1.19~1.85) | <0.001 | 1.39 (1.06~1.82) | 0.019 |  |
| Yes | Q1 | 67 | 4 (6) | 1(Ref) |  | 1(Ref) |  |  |
|  | Q2 | 95 | 10 (10.5) | 1.8 (0.56~5.74) | 0.32 | 1.84 (0.54~6.28) | 0.331 |  |
|  | Q3 | 69 | 9 (13) | 2.32 (0.72~7.55) | 0.16 | 1.85 (0.48~7.09) | 0.37 |  |
|  | Trend.test | 231 | 23 (10) | 1.48 (0.86~2.56) | 0.159 | 1.3 (0.7~2.41) | 0.414 |  |
| **Digestive tract anomaly** |  |  |  |  |  |  |  | 0.098 |
| No | Q1 | 518 | 31 (6) | 1(Ref) |  | 1(Ref) |  |  |
|  | Q2 | 584 | 38 (6.5) | 1.08 (0.67~1.74) | 0.741 | 1.15 (0.67~1.97) | 0.617 |  |
|  | Q3 | 583 | 59 (10.1) | 1.74 (1.13~2.69) | 0.013 | 1.6 (0.94~2.72) | 0.082 |  |
|  | Trend.test | 1685 | 128 (7.6) | 1.35 (1.08~1.68) | 0.008 | 1.28 (0.98~1.67) | 0.069 |  |
| Yes | Q1 | 116 | 3 (2.6) | 1(Ref) |  | 1(Ref) |  |  |
|  | Q2 | 49 | 6 (12.2) | 4.96 (1.24~19.82) | 0.024 | 1.58 (0.29~8.62) | 0.599 |  |
|  | Q3 | 52 | 11 (21.2) | 9.06 (2.53~32.49) | 0.001 | 3.63 (0.85~15.45) | 0.081 |  |
|  | Trend.test | 217 | 20 (9.2) | 2.76 (1.58~4.8) | <0.001 | 1.93 (0.92~4.02) | 0.081 |  |
| **ALI** |  |  |  |  |  |  |  | 0.911 |
| No | Q1 | 609 | 31 (5.1) | 1(Ref) |  | 1(Ref) |  |  |
|  | Q2 | 613 | 41 (6.7) | 1.32 (0.83~2.1) | 0.248 | 1.19 (0.71~2.01) | 0.505 |  |
|  | Q3 | 608 | 60 (9.9) | 2 (1.3~3.08) | 0.002 | 1.75 (1.05~2.91) | 0.031 |  |
|  | Trend.test | 1830 | 132 (7.2) | 1.42 (1.15~1.77) | 0.001 | 1.34 (1.03~1.72) | 0.027 |  |
| Yes | Q1 | 25 | 3 (12) | 1(Ref) |  | 1(Ref) |  |  |
|  | Q2 | 20 | 3 (15) | 1.36 (0.27~6.74) | 0.707 | 0.64 (0.03~15.47) | 0.784 |  |
|  | Q3 | 27 | 10 (37) | 3.65 (1~13.26) | 0.05 | 2.02 (0.13~32.6) | 0.621 |  |
|  | Trend.test | 72 | 16 (22.2) | 2.01 (1.05~3.86) | 0.035 | 1.76 (0.47~6.59) | 0.401 |  |

**Table S6 Subgroup analysis of the associations between phosphate levels and in-hospital death**

| **Subgroup** | **Variable** | **n.total** | **n.event_%** | **crude.OR_95CI** | **crude.P_value** | **adj.OR_95CI** | **adj.P_value** | **P.for.interaction** |
| --- | --- | --- | --- | --- | --- | --- | --- | --- |
| **Gender** |  |  |  |  |  |  |  | 0.065 |
| Male | Q1 | 380 | 24 (6.3) | 1(Ref) |  | 1(Ref) |  |  |
|  | Q2 | 397 | 39 (9.8) | 1.62 (0.95~2.74) | 0.076 | 1.62 (0.87~3.04) | 0.13 |  |
|  | Q3 | 387 | 43 (11.1) | 1.85 (1.1~3.12) | 0.02 | 1.55 (0.8~2.99) | 0.192 |  |
|  | Trend.test | 1164 | 106 (9.1) | 1.34 (1.04~1.72) | 0.022 | 1.22 (0.89~1.67) | 0.215 |  |
| Female | Q1 | 254 | 17 (6.7) | 1(Ref) |  | 1(Ref) |  |  |
|  | Q2 | 236 | 14 (5.9) | 0.88 (0.42~1.83) | 0.73 | 0.78 (0.32~1.92) | 0.593 |  |
|  | Q3 | 248 | 35 (14.1) | 2.29 (1.25~4.21) | 0.008 | 2.49 (1.17~5.32) | 0.019 |  |
|  | Trend.test | 738 | 66 (8.9) | 1.59 (1.16~2.2) | 0.004 | 1.68 (1.12~2.5) | 0.012 |  |
| **Premature** |  |  |  |  |  |  |  | 0.266 |
| No | Q1 | 401 | 28 (7) | 1(Ref) |  | 1(Ref) |  |  |
|  | Q2 | 385 | 37 (9.6) | 1.42 (0.85~2.36) | 0.183 | 1.18 (0.65~2.14) | 0.59 |  |
|  | Q3 | 385 | 62 (16.1) | 2.56 (1.6~4.09) | <0.001 | 2.11 (1.19~3.75) | 0.01 |  |
|  | Trend.test | 1171 | 127 (10.8) | 1.62 (1.28~2.05) | <0.001 | 1.48 (1.11~1.98) | 0.008 |  |
| Yes | Q1 | 233 | 13 (5.6) | 1(Ref) |  | 1(Ref) |  |  |
|  | Q2 | 248 | 16 (6.5) | 1.17 (0.55~2.48) | 0.688 | 1.81 (0.7~4.72) | 0.222 |  |
|  | Q3 | 250 | 16 (6.4) | 1.16 (0.54~2.46) | 0.705 | 1.32 (0.46~3.74) | 0.604 |  |
|  | Trend.test | 731 | 45 (6.2) | 1.07 (0.74~1.56) | 0.712 | 1.12 (0.68~1.83) | 0.655 |  |
| **CHD** |  |  |  |  |  |  |  | 0.756 |
| No | Q1 | 567 | 36 (6.3) | 1(Ref) |  | 1(Ref) |  |  |
|  | Q2 | 538 | 42 (7.8) | 1.25 (0.79~1.98) | 0.345 | 1.2 (0.68~2.1) | 0.526 |  |
|  | Q3 | 566 | 68 (12) | 2.01 (1.32~3.07) | 0.001 | 1.86 (1.09~3.18) | 0.024 |  |
|  | Trend.test | 1671 | 146 (8.7) | 1.44 (1.16~1.77) | 0.001 | 1.38 (1.05~1.81) | 0.02 |  |
| Yes | Q1 | 67 | 5 (7.5) | 1(Ref) |  | 1(Ref) |  |  |
|  | Q2 | 95 | 11 (11.6) | 1.62 (0.54~4.91) | 0.391 | 1.83 (0.55~6.15) | 0.326 |  |
|  | Q3 | 69 | 10 (14.5) | 2.1 (0.68~6.51) | 0.198 | 1.94 (0.52~7.27) | 0.325 |  |
|  | Trend.test | 231 | 26 (11.3) | 1.43 (0.83~2.46) | 0.198 | 1.36 (0.72~2.56) | 0.34 |  |
| **Digestive tract anomaly** |  |  |  |  |  |  |  | 0.232 |
| No | Q1 | 518 | 37 (7.1) | 1(Ref) |  | 1(Ref) |  |  |
|  | Q2 | 584 | 46 (7.9) | 1.11 (0.71~1.74) | 0.645 | 1.23 (0.72~2.09) | 0.453 |  |
|  | Q3 | 583 | 66 (11.3) | 1.66 (1.09~2.53) | 0.018 | 1.69 (0.99~2.89) | 0.053 |  |
|  | Trend.test | 1685 | 149 (8.8) | 1.31 (1.06~1.62) | 0.014 | 1.31 (1~1.71) | 0.048 |  |
| Yes | Q1 | 116 | 4 (3.4) | 1(Ref) |  | 1(Ref) |  |  |
|  | Q2 | 49 | 7 (14.3) | 4.67 (1.3~16.76) | 0.018 | 1.78 (0.34~9.39) | 0.494 |  |
|  | Q3 | 52 | 12 (23.1) | 8.4 (2.56~27.55) | <0.001 | 4.31 (1~18.66) | 0.05 |  |
|  | Trend.test | 217 | 23 (10.6) | 2.74 (1.59~4.71) | <0.001 | 2.09 (1~4.37) | 0.051 |  |
| **ALI** |  |  |  |  |  |  |  | 0.798 |
| No | Q1 | 609 | 38 (6.2) | 1(Ref) |  | 1(Ref) |  |  |
|  | Q2 | 613 | 49 (8) | 1.31 (0.84~2.03) | 0.234 | 1.29 (0.77~2.14) | 0.33 |  |
|  | Q3 | 608 | 68 (11.2) | 1.89 (1.25~2.86) | 0.003 | 1.77 (1.07~2.94) | 0.027 |  |
|  | Trend.test | 1830 | 155 (8.5) | 1.38 (1.12~1.7) | 0.002 | 1.33 (1.04~1.72) | 0.025 |  |
|  |  |  |  |  |  |  |  |  |
| Yes | Q1 | 25 | 3 (12) | 1(Ref) |  | 1(Ref) |  |  |
|  | Q2 | 20 | 4 (20) | 1.83 (0.36~9.35) | 0.466 | 1.27 (0.01~192.94) | 0.925 |  |
|  | Q3 | 27 | 10 (37) | 4.31 (1.03~18.15) | 0.046 | 16.37 (0.08~3261.27) | 0.301 |  |
|  | Trend.test | 72 | 17 (23.6) | 2.11 (1.04~4.29) | 0.039 | 5.82 (0.46~74.42) | 0.175 |  |

**Table S7 Subgroup analysis of the associations between phosphate levels and hospital LOS.**

| **Subgroup** | **Variable** | **n** | **crude.Coefficient_95CI** | **crude.P_value** | **adj.Coefficient_95CI** | **adj.P_value** | **P.for.interaction** |
| --- | --- | --- | --- | --- | --- | --- | --- |
| **Gender** |  |  |  |  |  |  | 0.845 |
| Male | Q1 | 380 | 0(Ref) |  | 0(Ref) |  |  |
|  | Q2 | 397 | -5.7 (-9.43~-1.97) | 0.003 | -4.26 (-8~-0.51) | 0.026 |  |
|  | Q3 | 387 | -5.39 (-9.15~-1.64) | 0.005 | -4.44 (-8.48~-0.4) | 0.032 |  |
|  | Trend.test | 1164 | -2.69 (-4.57~-0.81) | 0.005 | -2.23 (-4.25~-0.21) | 0.031 |  |
| Female | Q1 | 254 | 0(Ref) |  | 0(Ref) |  |  |
|  | Q2 | 236 | -2.3 (-6.57~1.97) | 0.291 | -3.44 (-7.73~0.86) | 0.117 |  |
|  | Q3 | 248 | -4.87 (-9.09~-0.65) | 0.024 | -4.37 (-8.9~0.16) | 0.059 |  |
|  | Trend.test | 738 | -2.44 (-4.54~-0.33) | 0.024 | -2.21 (-4.47~0.05) | 0.056 |  |
| **Premature** |  |  |  |  |  |  | 0.092 |
| No | Q1 | 401 | 0(Ref) |  | 0(Ref) |  |  |
|  | Q2 | 385 | -2.64 (-5.37~0.09) | 0.058 | -2.46 (-5.5~0.57) | 0.112 |  |
|  | Q3 | 385 | -6.53 (-9.26~-3.8) | <0.001 | -5.05 (-8.31~-1.79) | 0.002 |  |
|  | Trend.test | 1171 | -3.26 (-4.63~-1.9) | <0.001 | -2.52 (-4.15~-0.9) | 0.002 |  |
| Yes | Q1 | 233 | 0(Ref) |  | 0(Ref) |  |  |
|  | Q2 | 248 | -8.66 (-13.56~-3.76) | 0.001 | -6.63 (-12.55~-0.71) | 0.029 |  |
|  | Q3 | 250 | -4.74 (-9.63~0.15) | 0.058 | -3.14 (-9.43~3.15) | 0.328 |  |
|  | Trend.test | 731 | -2.29 (-4.75~0.16) | 0.068 | -1.48 (-4.64~1.67) | 0.356 |  |
| **CHD** |  |  |  |  |  |  | 0.754 |
| No | Q1 | 567 | 0(Ref) |  | 0(Ref) |  |  |
|  | Q2 | 538 | -4.07 (-7.13~-1.01) | 0.009 | -3.89 (-6.89~-0.88) | 0.011 |  |
|  | Q3 | 566 | -4.82 (-7.84~-1.8) | 0.002 | -4.7 (-7.87~-1.54) | 0.004 |  |
|  | Trend.test | 1671 | -2.41 (-3.92~-0.9) | 0.002 | -2.36 (-3.95~-0.78) | 0.003 |  |
| Yes | Q1 | 67 | 0(Ref) |  | 0(Ref) |  |  |
|  | Q2 | 95 | -6.57 (-13.76~0.62) | 0.075 | -5.22 (-13.33~2.89) | 0.208 |  |
|  | Q3 | 69 | -8.13 (-15.86~-0.39) | 0.041 | -3.69 (-12.89~5.5) | 0.432 |  |
|  | Trend.test | 231 | -4.05 (-7.91~-0.18) | 0.041 | -1.93 (-6.53~2.66) | 0.41 |  |
| **Digestive tract anomaly** |  |  |  |  |  |  | 0.271 |
| No | Q1 | 518 | 0(Ref) |  | 0(Ref) |  |  |
|  | Q2 | 584 | -5.79 (-8.83~-2.75) | <0.001 | -4.6 (-7.6~-1.6) | 0.003 |  |
|  | Q3 | 583 | -5.91 (-8.95~-2.87) | <0.001 | -4.24 (-7.45~-1.03) | 0.01 |  |
|  | Trend.test | 1685 | -2.9 (-4.42~-1.37) | <0.001 | -2.11 (-3.71~-0.5) | 0.01 |  |
| Yes | Q1 | 116 | 0(Ref) |  | 0(Ref) |  |  |
|  | Q2 | 49 | -1.18 (-8.97~6.61) | 0.767 | -3.85 (-12.66~4.97) | 0.393 |  |
|  | Q3 | 52 | -9.1 (-16.73~-1.47) | 0.02 | -11.27 (-20.62~-1.92) | 0.019 |  |
|  | Trend.test | 217 | -4.22 (-7.97~-0.48) | 0.028 | -5.48 (-10.09~-0.87) | 0.021 |  |
| **AKI** |  |  |  |  |  |  | 0.001 |
| No | Q1 | 623 | 0(Ref) |  | 0(Ref) |  |  |
|  | Q2 | 616 | -4.01 (-6.85~-1.16) | 0.006 | -3.44 (-6.28~-0.61) | 0.017 |  |
|  | Q3 | 604 | -4.81 (-7.67~-1.95) | 0.001 | -3.85 (-6.9~-0.8) | 0.014 |  |
|  | Trend.test | 1843 | -2.41 (-3.84~-0.98) | 0.001 | -1.95 (-3.47~-0.42) | 0.012 |  |
| Yes | Q1 | 11 | 0(Ref) |  | 0(Ref) |  |  |
|  | Q2 | 17 | -23.37 (-44.2~-2.53) | 0.032 | -28.17 (-47.05~-9.29) | 0.007 |  |
|  | Q3 | 31 | -21.26 (-40.15~-2.36) | 0.032 | -25.43 (-43.56~-7.3) | 0.01 |  |
|  | Trend.test | 59 | -8.54 (-17.73~0.64) | 0.073 | -11.31 (-20.71~-1.9) | 0.025 |  |
| **ALI** |  |  |  |  |  |  | <0.001 |
| No | Q1 | 609 | 0(Ref) |  | 0(Ref) |  |  |
|  | Q2 | 613 | -4.04 (-6.83~-1.26) | 0.004 | -3.33 (-6.09~-0.57) | 0.018 |  |
|  | Q3 | 608 | -3.88 (-6.67~-1.09) | 0.006 | -3.5 (-6.46~-0.54) | 0.021 |  |
|  | Trend.test | 1830 | -1.94 (-3.34~-0.55) | 0.006 | -1.77 (-3.25~-0.29) | 0.019 |  |
| Yes | Q1 | 25 | 0(Ref) |  | 0(Ref) |  |  |
|  | Q2 | 20 | -11.92 (-34.95~11.11) | 0.314 | -30.8 (-63.29~1.68) | 0.07 |  |
|  | Q3 | 27 | -35.26 (-56.57~-13.95) | 0.002 | -30.3 (-61.31~0.71) | 0.063 |  |
|  | Trend.test | 72 | -17.69 (-28.29~-7.09) | 0.002 | -12.98 (-28.17~2.21) | 0.102 |  |

**Table S8 Multivariate Cox proportional hazards regression analyses of serum phosphate levels and 28-day mortality (patients who died within 24 hours of admission, patients diagnosed with congenital hereditary diseases and tumour were excluded)**

| Variable | n.total | n.event_% | Model I | | Model Ⅱ | |
| --- | --- | --- | --- | --- | --- | --- |
|  |  |  | crude.HR (95%CI) | crude.P value | adj.HR (95%CI) | adj.P value |
| phosphate | 1821 | 114 (6.3) | 1.16 (0.96~1.39) | 0.116 | 1.13 (0.9~1.42) | 0.283 |
| Phosphate  tertiles | 1821 | 114 (6.3) |  |  |  |  |
| Q1 | 602 | 25 (4.2) | 1(Ref) |  | 1(Ref) |  |
| Q2 | 611 | 39 (6.4) | 1.55 (0.94~2.56) | 0.087 | 1.62 (0.92~2.87) | 0.096 |
| Q3 | 608 | 50 (8.2) | 2.04 (1.26~3.29) | 0.004 | 1.97 (1.11~3.49) | 0.021 |
| Trend.test |  |  | 1.41 (1.12~1.78) | 0.003 | 1.38 (1.05~1.81) | 0.022 |

Model I : no other covariates were adjusted.

Model Ⅱ: Adjustment factors included age,gender,premature, Neonatal asphyxia, AKI, ALI and Laboratory examination（Prealbumin, WBC, Platelets, Ck-MB, LAC）

**Table S9 Multivariate Cox proportional hazards regression analyses of serum phosphate levels and 28-day mortality (Patients who died within 24 hours after admission, those diagnosed with congenital hereditary diseases, tumour and AKI were excluded)**

| Variable | n.total | n.event_% | Model I | | Model Ⅱ | |
| --- | --- | --- | --- | --- | --- | --- |
|  |  |  | crude.HR (95%CI) | crude.P value | adj.HR (95%CI) | adj.P value |
| phosphate | 1765 | 106 (6) | 1.13 (0.93~1.38) | 0.23 | 1.11 (0.87~1.42) | 0.4 |
| Phosphate  tertiles | 1765 | 106 (6) |  |  |  |  |
| Q1 | 591 | 25 (4.2) | 1(Ref) |  | 1(Ref) |  |
| Q2 | 595 | 37 (6.2) | 1.48 (0.89~2.46) | 0.13 | 1.52 (0.85~2.71) | 0.157 |
| Q3 | 579 | 44 (7.6) | 1.84 (1.13~3.01) | 0.015 | 1.75 (0.97~3.15) | 0.065 |
| Trend.test |  |  | 1.34 (1.06~1.71) | 0.015 | 1.3 (0.98~1.73) | 0.069 |

Model I : no other covariates were adjusted.

Model Ⅱ: Adjustment factors included age, gender, premature, Neonatal asphyxia, ALI and Laboratory examination（Prealbumin, WBC, Platelets, Ck-MB, LAC）

**Table S10 Multivariate logistic regression analyses of phosphate level and in- hospital death (patients who died within 24 hours of admission, patients diagnosed with congenital hereditary diseases and tumour were excluded)**

| Variable | n.total | n.event_% | Model I |  | Model Ⅱ |  |
| --- | --- | --- | --- | --- | --- | --- |
|  |  |  | crude.OR (95%CI) | crude.P value | adj.OR (95%CI) | adj.P value |
| phosphate | 1821 | 141 (7.7) | 1.15 (0.97~1.38) | 0.112 | 1.17 (0.93~1.47) | 0.18 |
| Phosphate  tertiles |  |  |  |  |  |  |
| Q1 | 602 | 33 (5.5) | 1(Ref) |  | 1(Ref) |  |
| Q2 | 611 | 48 (7.9) | 1.47 (0.93~2.32) | 0.099 | 1.55 (0.91~2.64) | 0.109 |
| Q3 | 608 | 60 (9.9) | 1.89 (1.22~2.93) | 0.005 | 1.91 (1.12~3.28) | 0.018 |
| Trend.test | 1821 | 141 (7.7) | 1.37 (1.1~1.69) | 0.005 | 1.37 (1.05~1.78) | 0.019 |

Model I : no other covariates were adjusted.

Model Ⅱ: Adjustment factors included age, gender, premature, Neonatal asphyxia, AKI, ALI and Laboratory examination（Prealbumin, WBC, Platelets, Ck-MB, LAC)

**Table S11 Multivariate logistic regression analyses of phosphate level and in- hospital mortality(Patients who died within 24 hours after admission, those diagnosed with congenital hereditary diseases, tumour and AKI were excluded)**

| Variable | n.total | n.event_% | Model I |  | Model Ⅱ |  |
| --- | --- | --- | --- | --- | --- | --- |
|  |  |  | crude.OR (95%CI) | crude.P value | adj.OR (95%CI) | adj.P value |
| phosphate | 1765 | 133 (7.5) | 1.13 (0.94~1.36) | 0.194 | 1.14 (0.89~1.44) | 0.296 |
| Phosphate  tertiles |  |  |  |  |  |  |
| Q1 | 591 | 33 (5.6) | 1(Ref) |  | 1(Ref) |  |
| Q2 | 595 | 46 (7.7) | 1.42 (0.89~2.25) | 0.14 | 1.48 (0.86~2.53) | 0.154 |
| Q3 | 579 | 54 (9.3) | 1.74 (1.11~2.73) | 0.016 | 1.71 (0.98~2.98) | 0.057 |
| Trend.test | 1765 | 133 (7.5) | 1.31 (1.05~1.63) | 0.016 | 1.3 (0.99~1.7) | 0.06 |

Model I : no other covariates were adjusted.

Model Ⅱ: Adjustment factors included age, gender, premature, Neonatal asphyxia, ALI and Laboratory examination（Prealbumin, WBC, Platelets, Ck-MB, LAC)

**Table S12**  **Multivariate linear regression analyses of phosphate level and in- hospital LOS (Patients who died within 24 hours after admission, those diagnosed with congenital hereditary diseases, tumour and AKI were excluded)**

| Variable | n | Model I |  | Model Ⅱ |  |
| --- | --- | --- | --- | --- | --- |
|  |  | crude.Coefficient (95%CI) | crude.P value | adj.Coefficient (95%CI) | adj.P value |
| phosphate | 1632 | -1.33 (-2.72~0.06) | 0.061 | -1.29 (-2.86~0.29) | 0.109 |
| Phosphate  tertiles |  |  |  |  |  |
| Q1 | 558 | 0(Ref) |  | 0(Ref) |  |
| Q2 | 549 | -4.41 (-7.41~-1.41) | 0.004 | -3.82 (-6.79~-0.85) | 0.012 |
| Q3 | 525 | -4.49 (-7.53~-1.45) | 0.004 | -4.12 (-7.34~-0.9) | 0.012 |
| Trend.test | 1632 | -2.27 (-3.79~-0.75) | 0.003 | -2.09 (-3.7~-0.48) | 0.011 |

Model I : no other covariates were adjusted.

Model Ⅱ: Adjustment factors included age, gender, premature, Neonatal asphyxia, Sepsis and Digestive tract anomaly and Laboratory examination (Hemoglobin, WBC, Platelet, Ck-MB, BUN, Prealbumin, LAC)
